# *QuickStats:* Percentage* of Adults Aged 18–64 Years with a Usual Place for Health Care,^†^ by Race/Ethnicity^§^ — National Health Interview Survey, United States, 2008 and 2018

**DOI:** 10.15585/mmwr.mm6905a6

**Published:** 2020-02-07

**Figure:**
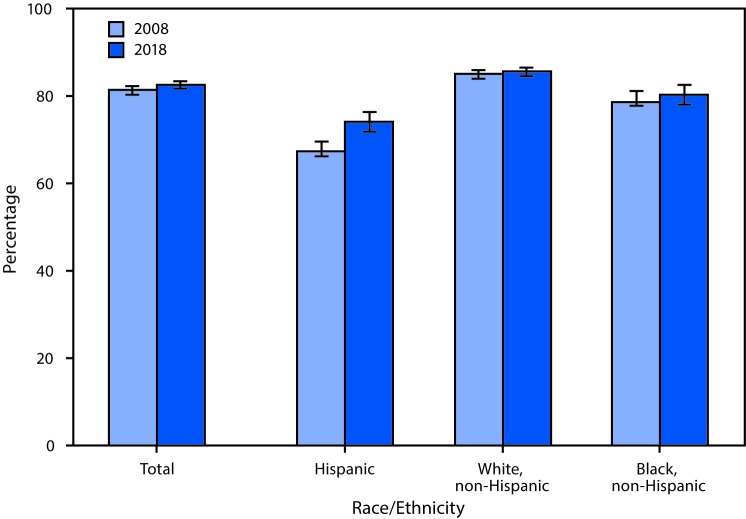
Although the percentage of Hispanic adults aged 18–64 years who had a usual place to go for medical care was higher in 2018 (74.1%) than in 2008 (67.3%), Hispanic adults remained the least likely to have a usual place to go for medical care. Non-Hispanic white adults were the most likely to have a usual place for medical care in both 2008 (85.0%) and 2018 (85.5%). In 2008, 78.7% of non-Hispanic black adults had a usual place for health care compared with 80.4% in 2018.

**Source:** National Health Interview Survey, 2008 and 2018 data. https://www.cdc.gov/nchs/nhis.htm.

